# Tracing chronological shifts in farmland demarcation trees in southwestern Japan: implications from species distribution patterns, folk nomenclature, and multiple usage

**DOI:** 10.1186/s13002-019-0301-8

**Published:** 2019-04-27

**Authors:** Yoshinori Tokuoka, Fukuhiro Yamasaki, Kenichiro Kimura, Kiyokazu Hashigoe, Mitsunori Oka

**Affiliations:** 1 Division of Biodiversity, Institute for Agro-Environmental Sciences, National Agriculture and Food Research Organization, 3-1-3, Kannondai, Tsukuba, Ibaraki 305-8604 Japan; 2 Genetic Resources Center, National Agriculture and Food Research Organization, 2-1-2, Kannondai, Tsukuba, Ibaraki 305-8602 Japan; 3 Rural Development Division, Japan International Research Center for Agricultural Sciences, 1-1, Ohwashi, Tsukuba, Ibaraki 305-8686 Japan; 4 1-8-37-401, Iwasakimachi, Matsuyama, Ehime, 790-0854 Japan; 5 Research Institute, Tokyo University of Agriculture, 1-1-1, Sakuragaoka, Setagaya, Tokyo, 156-8502 Japan

**Keywords:** Agricultural heritage, Agrobiodiversity, Cultural landscape, Ethnobiological linguistics, Floristic composition, Vernacular names, Traditional knowledge

## Abstract

**Background:**

Understanding the history of anthropogenic vegetation is often difficult due to the lack of tangible historical evidence. In this study, we examined chronological changes of farmland demarcation trees planted on alluvial plains along the Hijikawa River in southwestern Japan based on species distribution patterns, folk nomenclature, and multiple usage of the trees.

**Methods:**

The species composition of demarcation trees was investigated at 47 sites in 13 villages. We performed hierarchical clustering using Bray–Curtis measures to detect groups of similar tree composition and permutational multivariate analysis of variance to test whether differences in species composition correspond to village units. To better understand the traditional knowledge of demarcation trees, we conducted interviews with 53 farmers, most of whom were over 60 years old.

**Results:**

Clustering resulted in six tree composition groups. The group characterized by the most frequently planted species, *Chaenomeles speciosa*, dominated around lower reach villages. The group characterized by *Euonymus japonicus* dominated around middle reach villages, and that characterized by *Salix pierotii* was mainly located around upper reach villages. *Chaenomeles speciosa* was always identified with the standard Japanese name *boke* or similar names. *Euonymus japonicus* and several other species were also called *boke* by many farmers. Several elderly farmers stated that *C. speciosa* was pervasive in upper and middle reach villages in their youth, suggesting the prototypical use of *C. speciosa* in the study area. In addition, some minor species were likely to have been left after commercial crop production or subsistence use between the late nineteenth and mid-twentieth centuries, including *Morus* sp. and *Celtis sinensis* for sericulture, *Salix koriyanagi* for fiber production, and *Gardenia jasminoides* for food coloration. The name *kōshin bana* recorded for *E. japonicus* suggests that the species’ use originated from the folk faiths *Kōshin-shinkō* and/or *Shōmen-Kongō.*

**Conclusions:**

The composition of demarcation trees in the region has not been stable over time, but instead changed to reflect the local livelihood, industry, and faiths. Despite the lack of tangible historical evidence, the spatial distribution patterns, folk nomenclature, and traditional knowledge of plants can provide clues to trace the chronological background of ecotopes in anthropogenic landscapes.

## Background

The socio-ecological production landscapes and seascapes are important to support the livelihoods of people and protect global biodiversity [1, 2]. For example, non-crop anthropogenic vegetation in agricultural areas, such as hedgerows, groves, and isolated trees, creates habitats for diverse organisms [3], supports crop production by nurturing natural enemies and pollinators and protecting against soil erosion and nutrient runoff [4], provides nutrients to the soil [5, 6], and adds to the esthetics of rural landscapes [7]. The chronological changes of anthropogenic vegetation throughout history may be successfully traced when old maps and/or documents are available, as in the case of hedgerows in the UK [8]. However, no such documents exist for many landscapes.

Isolated woody plants have been preserved in farmland areas of various agricultural landscapes for many purposes. For example, such plants were maintained in Australia as paddock trees to provide shelter for pastures, crops, and livestock and to protect soils [3, 9]. Woody plants in agricultural areas of Japan were used to dry harvested rice and to collect persimmon tannin [10, 11] and in Kenya to collect tree products such as medicinal latex and timber [12, 13]. In Lao PDR and Thailand, trees were maintained to provide shade for humans and livestock and to obtain plant materials for construction, furniture, tools, foods, medicine, fodder, fertilizer, fuel, resin, and tanning, among other uses [14–17], as well as to secure rice yields in dry years [16]. In Bangladesh, trees were planted for cash crops and subsistence non-timber forest products and to maintain soil fertility [18]. In addition to these uses with some direct or indirect economic value, isolated woody plants including heavily trimmed trees and/or shrubs were traditionally maintained to demarcate farmland boundaries in many places, including Japan [19] and Kenya [12]. These plants are primarily maintained as markers for land ownership or to easily restore farmland boundaries after flooding. Fragmentary information from old documents noted the use of farmland demarcation trees in the seventeenth century in eastern Japan [20] and in the early twentieth century in Kenya [21]. Notes in a novel [22] suggest that such trees were also used in both paddy and upland fields in eastern Japan in the late nineteenth century. In interviews with farmers aged 70 to 80 years in Ibaraki Prefecture, the farmers were unable to identify the age of such isolated tree markers near their farms [23], suggesting that such markers can generally survive for more than half a century. With regard to the historical background of farmland demarcation trees in Japan, however, it remains unclear which species are traditional choices and the reason(s) for those species to be used.

Language reflects how local people recognize and utilize the natural setting and resources around them [24–26]. Thus, when studying agricultural history, it is important to combine folk plant nomenclature along with ethnobiology, ecology, and/or genetics of local flora. Those approaches have been used to elucidate agroecological historical information such as the domestication process of crops [27], genetic diversity of crop varieties [28], and the cultural diffusion process of plant usage [29, 30] and artifacts [31]. Even if no tangible historical evidence is available, research approaches relying on folk plant nomenclature and other plant information make it feasible to trace the history of plant use in many regions around the world.

On the alluvial plains along the Hijikawa River in Ehime Prefecture, southwestern Japan, isolated woody plants have been maintained to demarcate farmland boundaries. The landscape containing demarcation trees in the villages of Gorou and Wakamiya on the Hijikawa River was designated as an important cultural landscape in Japan by the Agency of Cultural Affairs [32]. The agency reported the landscape was characterized by the presence of demarcation tree species such as *Chaenomeles speciosa, Euonymus japonicus*, and *Salix* spp. However, the historical background of the choice of these tree species and whether the two designated villages preserve the most typical floristic diversity of farmland boundary trees in the region remain unclear. Understanding the historical reasons why local people selected each tree species would add to our appreciation of this anthropogenic vegetation and landscape. In addition, if there are clear compositional differences in demarcation tree species among the villages along the river, approaches to conserving the cultural landscape should be modified. Therefore, in this study, we aimed to trace the history of farmland demarcation trees in villages along the Hijikawa River by focusing on the species distribution patterns, folk nomenclature, and multiple uses of the trees.

## Material and methods

### Study area

**Fig. 1:**
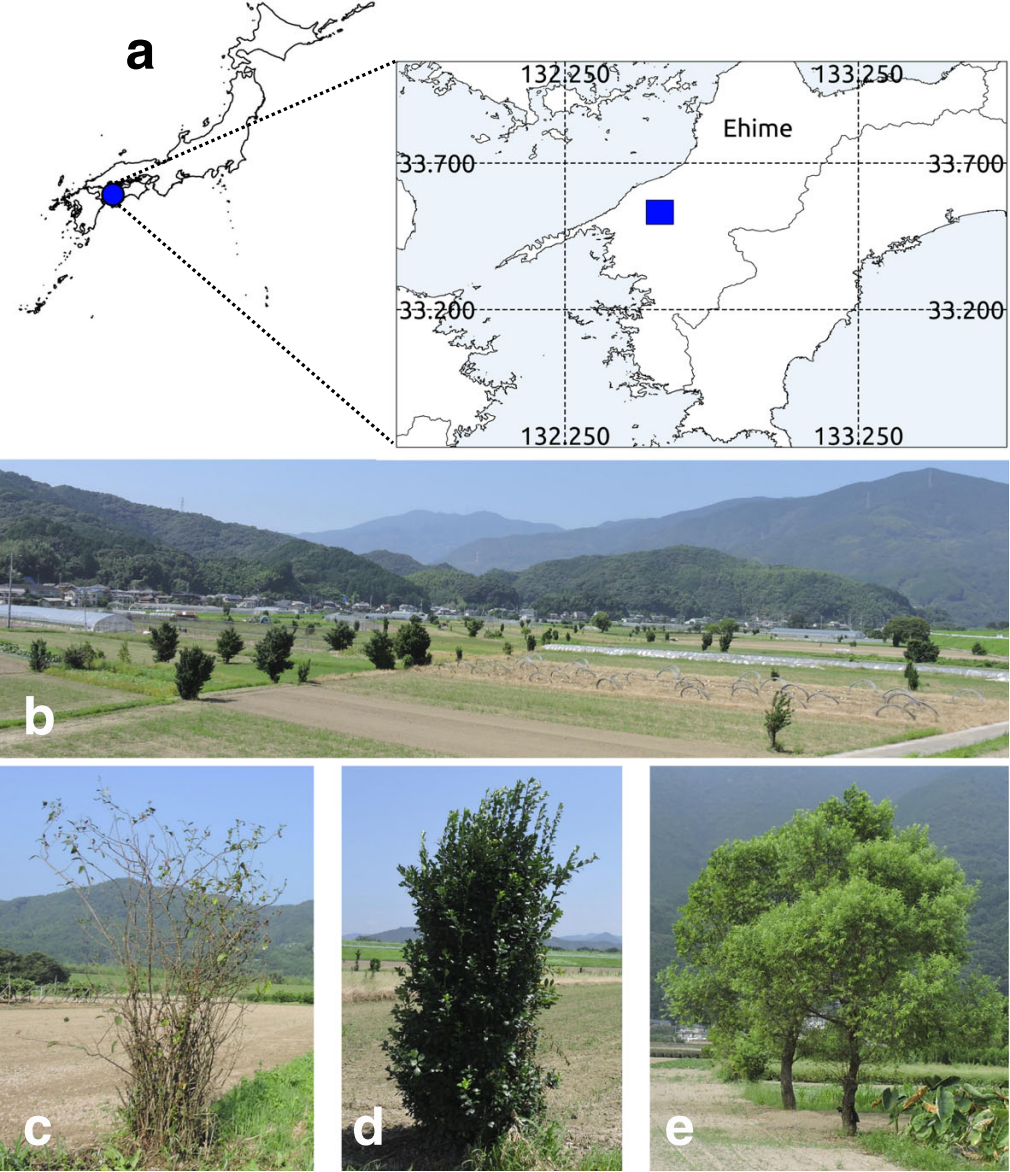
Study site location, scenery, and examples of three dominant demarcation trees. **a** Maps of the study site. **b** Demarcation trees in upland fields in the Gorou area: **c** *Chaenomeles speciosa*, **d** *Euonymus japonicus*, and **e** *Salix pierotii*

Farmland demarcation trees were investigated in the area along the Hijikawa River in Ozu city, Ehime Prefecture (Fig. 1). According to the 1981–2010 statistics recorded at the Ozu weather station, the mean temperature was 15.6 °C and mean annual rainfall was 1648.8 mm. Upland fields and irrigated paddy fields predominate on the alluvial plains. The middle to lower reaches of the Hijikawa River follow a northwestern course to where the river flows into the Seto Inland Sea. According to a history of the area [33], countermeasures against flooding have long been an important mission of local authorities. Several centuries ago in the Edo period, to reduce the flow rate during flash flooding events, bamboos species such as *Phyllostachys aurea*, *Phyllostachys edulis*, *Phyllostachys reticulata*, and *Pleioblastus simonii* and tall trees such as *Aphananthe aspera* and *Celtis sinensis* in mixed plantings with *P. aurea* or *P. simonii* were planted along the river bank. Moreover, stone walls were constructed to reduce the flow rate or to deflect the flow to some parts of the shore. Although Kanogawa Dam and Nomura Dam were constructed in 1959 and 1982, respectively, their ability to control heavy rain fall remains imperfect. Even after the dam construction, flooding events have still damaged the residential and farmland areas along the lower to upper reaches of the Hijikawa River in Ozu city. Under such environmental conditions, it is presumed that demarcation trees have been necessary in the region to restore farmland boundaries after flooding for centuries. However, no historical documents on the farmland demarcation trees have been found so far.

Farmland use on the alluvial plains in the study areas has been wet rice paddy and arable fields. According to Ozu-shishi-hensan-kai [33], the main crops were paddy rice, wheat, barley, and various cereals and vegetables in the late nineteenth century. The main crops of arable fields have been changing since the mid-nineteenth century, reflecting market trends. Sericulture began to be an important commercial activity in the mid-nineteenth century, hit peak production in the early twentieth century, and fell into a severe decline in the mid-twentieth century. Commercial *S. koriyanagi* cultivation for fiber production in Ehime Prefecture began in the early twentieth century, hit peak production in the 1930s, and declined in the 1960s [34]. The period of severe decline was the same in Ozu city [35].

A series of aerial photographs after WWII provided by the Geospatial Information Authority of Japan, which are available online (cyberjapandata.gsi.go.jp), indicated that land consolidation occurred in some farmland areas on the alluvial plains in Ozu city. In those areas, the farmland patches became neat squares demarcated by linear field ridges or artificial marker piles. In the rest of the areas, the farmland patches remain small and the demarcation trees are still maintained mostly on the boundaries of ridge-less arable fields.

### Marker sampling

Our preliminary observations revealed demarcation trees on the alluvial plains between Udzu village on the upper reach and Shirataki village on the lower reach of the Hijikawa River. To sample farmland boundaries in the area systematically, we adopted a protocol that was modified from that of Tokuoka and Hosogi [23]. First, equidistant cells (each 100 m × 100 m) were overlaid on a map of the area. Then, half of the cells (i.e., 48 of 96) were randomly chosen. In each chosen cell, we visited five farmland boundaries marked with isolated woody plants that were closest to the centroid point of the cell. The species of each marker plant was identified in the field, or the plant was photographed, or branches and leaves were sampled for later species identification. At two points in Sugeta village and one point in Tada village, only four boundaries with demarcation trees were found, but these three points were still included in the following analysis. At the point in Shirataki village located at the lowermost area, only one *Celtis sinensis* was found as a marker, and the data from that cell were omitted in the following analysis. Consequently, a total of 47 cells were surveyed from July 18 to August 18 in 2016. In this paper, plant nomenclature follows Yonekura and Kajita [36] and plant species origin and leaf types follow Miyawaki et al. [37] and Satake [38]. Dried specimens of sampled plants were deposited at the Ehime Prefectural Museum.

The locations (longitude and latitude) of the boundary markers were recorded in JGD2000 by comparing the positions in the fields and those on the recent photographs taken by the Geospatial Information Authority of Japan. For the map illustration using Quantum GIS software version 2.14 [39], mean longitude and latitude of surveyed markers around the centroid of each chosen cell were used as the representative point. Village section data were obtained from Shobunsha [40], and water area data were obtained from the Geographical Survey Institute [41].

### Interviews

Semi-structured interviews were conducted in the field with 53 farmers working close to the point of marker sampling from July 18 to August 18 in 2016. The informants were 36 males, 14 females, and 3 couples. Age groups of the informants were as follows: 40s, *n* = 2; 50s, *n* = 2; 60s, *n* = 14; 70s, *n* = 20; 80s, *n* = 14; and 90s, *n* = 1. In each interview, the following predetermined items were asked: the local name of the marker species that was present at the informant’s or neighboring fields, the reason for the species choice, whether there are uses other than demarcation, who introduced the plants and when, the means of planting, and the management method and frequency. Additional information provided during the interview was also recorded. The local names of the marker species are here given in italic font.

## Statistical analysis

The degree of compositional similarity of marker species among the 47 locations was inspected by hierarchical clustering [42]. The matrix used in this analysis consists of 47 rows (locations) and 24 columns (marker species), and each cell was filled with the number of individuals of each species relative to the total of all individuals of all species at each study location. Using this matrix, dissimilarity indices based on the Bray–Curtis measure were calculated and hierarchical clustering was performed with complete linkage. To test whether the different village identities correspond to the compositional dissimilarities of marker species, permutational multivariate analysis of variance (PERMANOVA [43]) using the same matrix was performed with 10,000 permutations.

These analyses were conducted with the vegan (https://cran.r-project.org/web/packages/vegan/index.html) and gplots (https://cran.r-project.org/web/packages/gplots/index.html) packages using R software version 3.3.3 [44].

## Results

### Spatial distribution of the marker plants

**Fig. 2:**
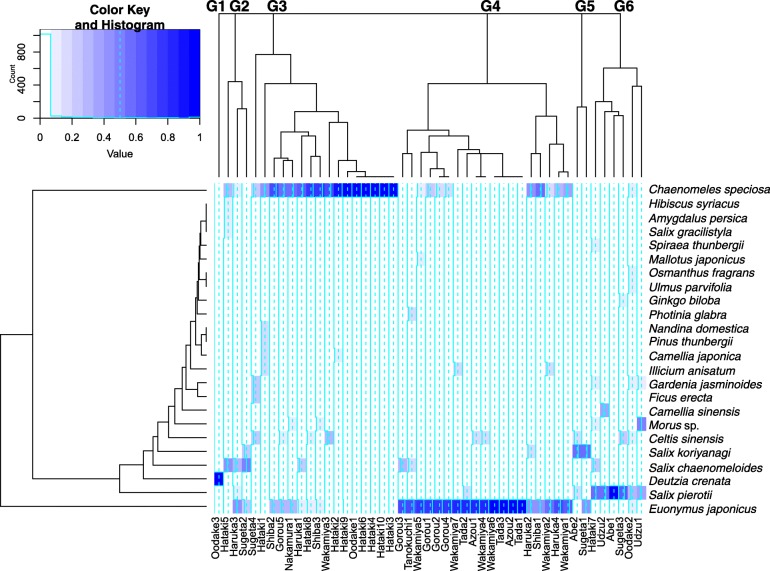
Dendrogram of woody species planted as demarcation trees at 47 survey locations. The six marker composition groups (G1–G6), which were determined by cutting this dendrogram at the minimum level of branching, correspond to the numbers labeled in Fig. 3

**Fig. 3:**
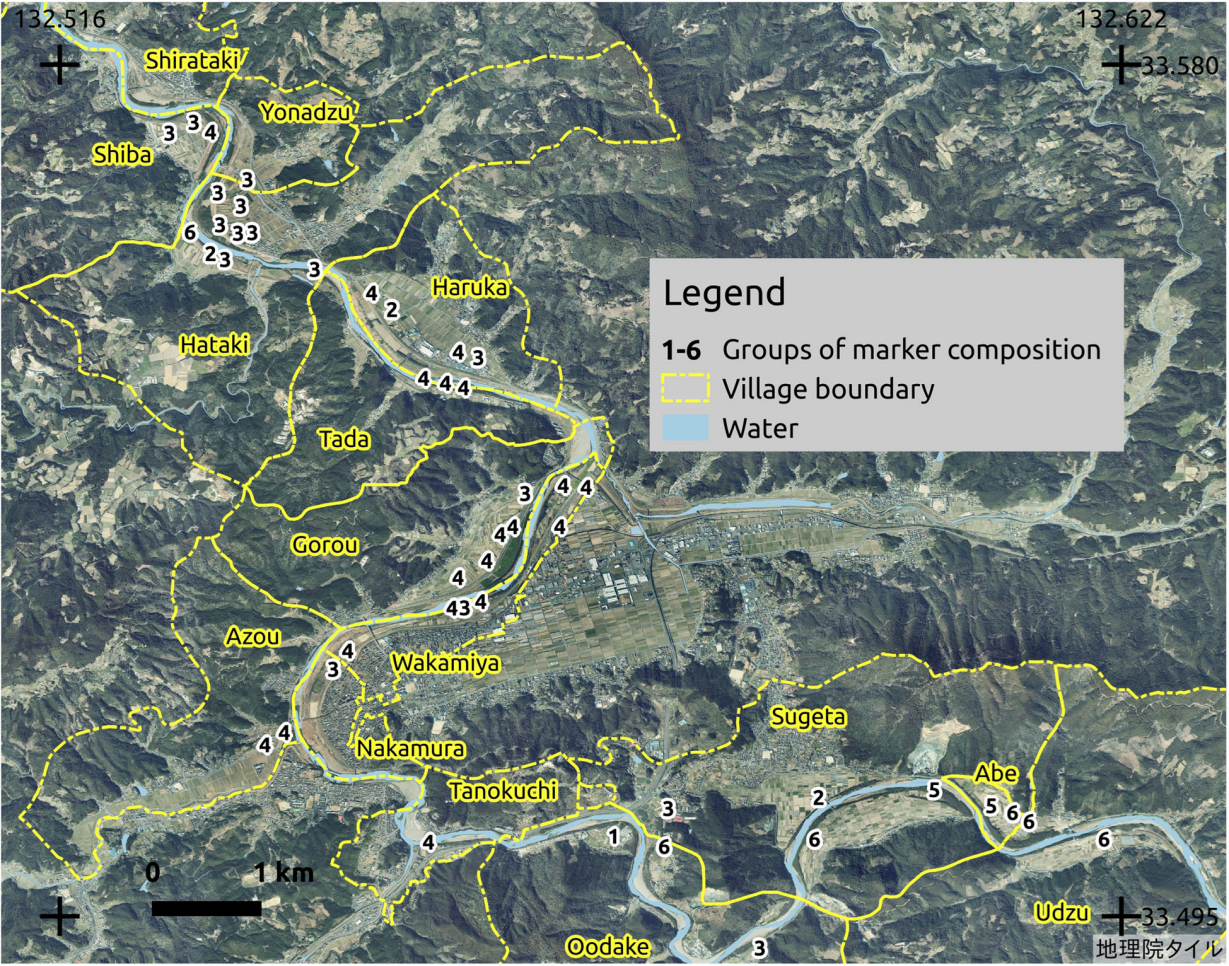
Distribution of the six marker composition groups used as demarcation trees along the Hijikawa River, Ozu City, Ehime Prefecture. The recent aerial photograph was obtained from the Geospatial Information Authority of Japan (Chiriin-chizu; https://maps.gsi.go.jp/)

A total of 415 individuals of 24 woody species (including one genus level identification of *Morus* sp.) were recorded (Table 1). According to the hierarchical clustering, six marker composition groups were recognized at the minimum level of pruning of the dendrogram (Fig. 2). Each group was characterized by a single dominant species: group 1, *Deutzia crenata* (*n* = 1); group 2, *Salix chaenomeloides* (*n* = 3); group 3, *Chaenomeles speciosa* (*n* = 16); group 4, *Euonymus japonicus* (*n* = 19); group 5, *Salix koriyanagi* (*n* = 2); and group 6, *Salix pierotii* (*n* = 6). According to PERMANOVA, marker composition differed significantly among villages (*F* = 3.78, *P* < 0.001). As shown in Fig. 3, group 3 (*C. speciosa* dominated) was mainly located around lower reach villages (Hataki and Shiba) and group 4 (*E. japonicus* dominated) was mainly located around middle reach villages (between Tanokuchi and Haruka and Tada). Group 6 (included *S. pierotii*) was often observed around upper reach villages (between Udzu and Oodake). It was difficult to draw any conclusions about the core distribution areas of groups 1, 2, and 5 because of their limited numbers of clustered points.

**Table 1:** Observed number of individuals, local name, species origin, and morphology of demarcation tree species along the Hijikawa River

Species	Observed number of individuals (% of total)	Local name (responses/total answers)	Species origin	Morphology
*Chaenomeles speciosa*	159 (38.3)	Boke* (24/29), *iga boke* (3/29), *bara boke* (1/29), *boke noki* (1/29)	A	D, T
*Euonymus japonicus*	136 (32.8)	*Boke* (8/28), DN (7/28), masaki* (4/28), *mayumi* (3/28), F (3/28), *kōshin bana* (1/28), *mame shiba* (1/28), *aoki* (1/28)	N	E
*Salix pierotii*	38 (9.2)	*Yanagi* (5/5)	N	D
*Salix chaenomeloides*	19 (4.6)	*Yanagi* (1/2), DN (1/2)	N	D
*Celtis sinensis*	12 (2.9)	Enoki* (1/1)	N	D
*Salix koriyanagi*	9 (2.2)	*Yanagi* (2/2)	A	D
*Deutzia crenata*	8 (1.9)	*Boke* (1/3), utsugi* (1/3), DN (1/3)	N	D
*Morus* sp.	7 (1.7)	*Kuwa* (2/3), *kuwa noki* (1/3)	A or N	D
*Camellia sinensis*	6 (1.4)		A	E
*Gardenia jasminoides*	4 (1.0)	Kuchinashi* (3/4), *boke* (1/4)	N	E
*Illicium anisatum*	3 (0.7)		N	E
*Camellia japonica*	2 (0.5)		N	E
*Mallotus japonicus*	1 (0.2)		N	D
*Ulmus parvifolia*	1 (0.2)		N	D
*Ginkgo biloba*	1 (0.2)		A	D
*Ficus erecta*	1 (0.2)		N	D
*Photinia glabra*	1 (0.2)		N	E
*Osmanthus fragrans*	1 (0.2)	Kimmokusei* (1/1)	A	E
*Pinus thunbergii*	1 (0.2)		N	E
*Amygdalus persica*	1 (0.2)		A	D
*Hibiscus syriacus*	1 (0.2)	*Boke* (2/2)	A	D
*Nandina domestica*	1 (0.2)		N	E
*Salix gracilistyla*	1 (0.2)		N	D
*Spiraea thunbergii*	1 (0.2)		A	D

Asterisks indicate the standard Japanese name of the plant species. DN indicates that the informant did not know the plant name. F indicates that the informant forgot the plant name

*A* alien species, *N* native species, *D* deciduous, *E* evergreen, *T* thorny

### Folk nomenclature

As shown in Table 1, local names were recorded for 11 species. The folk nomenclature of *C. speciosa* corresponds well to Western biological nomenclature because 82.8% of respondents (24/29) answered with the standard Japanese name of the species (*boke*) and the others all included it in the binomial names. Much fewer respondents recognized another dominant species, *E. japonicus*. Only 14.3% (4/28) answered with the standard Japanese name (*masaki*), and 35.7% (10/28) either did not know or forgot the name (DN and F in Table 1), 28.6% (8/28) used the name of *C. speciosa* (*boke*), and 10.7% (3/28) gave the standard name of *Euonymus sieboldianus* (*mayumi*). One male informant, who moved from the mountain area of Hataki village to Wakamiya village, called the plant *kōshin bana*, the name for *E. japonicus* used as hedges around his home. According to Tokiwai [45], old generations in the Miyoshi area including Tada and Haruka villages called some farmland demarcation trees *kōshin shiba*. Although it was not possible to examine the name corresponding to a particular species or higher taxon, the photograph of *kōshin shiba* in the reference included *E. japonicus* as an example. *Kōshin-shinkō* is a folk faith in Japan with Taoist origins, influenced by Shinto, Buddhism, and other local beliefs [46].

The standard name of *C. speciosa* (*boke*) was also used for *D. crenata*, *Gardenia jasminoides, Hibiscus syriacus*, and *Salix* sp. Although two informants used the binomial *yanagi boke* for some *Salix* individuals, we could not identify the *Salix* species that the informants were referring to. Therefore, the data were not included in Table 1. Thus, *boke*, the standard Japanese name of *C. speciosa*, seems to be the representative folk generic name for demarcation plants for many farmers in the region. Likewise, *yanagi* is the folk generic name corresponding to the genus *Salix* and *kuwa* corresponds to *Morus*.

### Introduction, management, and multiple usage

According to 51 of the 53 respondents, the introduction periods of demarcation trees were unknown because ancestors planted them. Five respondents stated that stones, rather than trees, were sometimes used as demarcation markers.

Forty-seven responses were obtained regarding the management of demarcation trees: 37 farmers pruned the trees whenever it becomes necessary, 3 said the season of pruning was fall or winter, 7 answered that they did not know the exact management practice, and 2 said when the owners of a focal demarcation trees agreed to prune them.

Reflecting the composition and abundance of demarcation trees shown in Table 1, most of the 53 responses regarding usages and management were for several dominant species. Many informants (73.4%, *n* = 39) answered that the demarcation trees had no alternative uses. Some informants stated that they or local people appreciated the fresh flowers or the plant of *C. speciosa* (*n* = 2) and *G. jasminoides* (*n* = 1) and noted the potential use of *C. speciosa* (*n* = 2) and *E. japonicus* (*n* = 1) for garden or hedge plants. The fruit of *G. jasminoides* had been used by local people as a food colorant in the past (*n* = 2). *Euonymus japonicus* had been used as pillars for stretching ropes and as marks for deciding cropping positions (*n* = 1). Some informants (9.4%, *n* = 5) stated that local florists or other outsiders have come to collect fresh flowers (maybe without any charge) for the past half century. Although no usage was mentioned for the *Salix* trees, one informant in Sugeta village stated the tree might be preferred because it grows tall and provides shade for resting, whereas another informant in Hataki village stated that the tall height of *Salix* trees is problematic for cropping.

### Marker replacement

Several respondents stated that they, their partners, or adjacent land owners replanted trees mostly by using cuttings when demarcation trees died by wilting. Those replanted trees included *Salix* individuals (*n* = 5), *E. japonicus* (*n* = 3), *C. speciosa* or *E. japonicus* (*n* = 1), *D. crenata* (*n* = 1), and *Osmanthus fragrans* (*n* = 1). According to two respondents, trees were planted at the position where land owners sharing a focal boundary agreed to plant. According to an informant in his 70s in Wakamiya village, *C. speciosa* has been gradually replaced with *E. japonicus* over the past several decades. Another informant in his 60s in the same village also remarked on the ease of planting *E. japonicus* by using cuttings*.* Nine informants ranging in age from their 60s to 80s in the upper reach villages of Udzu, Abe, and Sugeta stated that *C. speciosa* and/or *G. jasminoides* were common demarcation trees when they were young.

Some of farmland boundary trees have been maintained even after commercial crop production declined. The leaves of *Morus* sp. were fed to silkworm (*n* = 5), branches of *Celtis sinensis* were used for the cocooning of silkworm (*n* = 1), and *S. koriyanagi* was cultivated for fiber production (*n* = 1).

## Discussion

### Prototypicality of *C. speciosa*

Our findings suggest that *C. speciosa* has been the prototypical demarcation tree in the study region. The name *boke*, which is the standard Japanese name of *C. speciosa*, was widely used as the representative generic name for several demarcation tree species. The statements by local farmers about marker replacement in recent decades in Wakamiya village, located in the middle reach area, and the common use of *C. speciosa* in past decades in upper reach villages also support the prototypicality of *C. speciosa*. This alien Chinese species is thorny and bears edible fruits. The first record of *C. speciosa* in Japan (written as *moke*) was found in the document Honzou-Wamyou written around 918 [47]. Although the life forms and morphologies of several plants sharing the name *boke* were clearly different, such as the presence/absence of thorns and evergreen or deciduous leaves (Table 1), there was no naming distinction made by local people for those plants. This implies that locals are not paying close attention to these plants, perhaps because demarcation trees do not tend to serve multiple uses. Such folk nomenclature patterns for less useful plants are a well-known phenomenon in ethnobotany [25]. Demarcation trees in a paddy field landscape in central Japan served multiple uses [10], but the demarcation trees in an upland field landscape in eastern Japan [23] were generally used only for demarcation as observed in the present study.

### Possible influence of folk faiths on *E. japonicus*

Although folk nomenclature patterns and statements by local people suggested the prototypicality of *C. speciosa*, *E. japonicus* cannot be simply viewed as a latecomer. One informant gave its local name as *kōshin bana* (*bana* means “flower” in Japanese), which was used as a hedge plant at his birthplace homestead. Tokiwai [45] noted that some woody plants were called *kōshin shiba* (*shiba* means “bushy tree plants” in Japanese) and were used as demarcation trees around Hataki villages. In addition, use of the name *kōshin shiba* for *E. japonicus* was also recorded in the Hata region in southwestern Shikoku [48], which is adjacent to our study site. The folk faith *Shōmen-Kongō*, which was initially derived from Taoism and evolved from *Kōshin-shinkō* in Japan [46], might also be related to the use of *E. japonicus*. According to a local historian, Mr. Yasumasa Doi, the deity of the *Shōmen-Kongō* faith is believed to prevent and relieve flooding damages in some neighboring areas of this study region (personal communication). He also stated that people in these areas decorate statues of *Shōmen-Kongō* with *E. japonicus* as a local folk habit. A female informant who was over 90 years old stated that *Kōshin* meetings were held periodically before WWII, but the faith was no longer practiced after the war. The topography of the study area makes it prone to irregular flooding, suggesting that the folk faiths *Kōshin-shinkō* and/or *Shōmen-Kongō* might have had a possible linkage to the origin of *E. japonicus* use for demarcation trees*.*

### Use of similar plants for farmland demarcation trees, hedges, and gardening plants

As described in poems written around the eighth century [49], *D. crenata* is also a common and traditional hedge plant in Japan. Growing thorn hedges is an old practice in Japan, as depicted in old waka poems written around the fourteenth century, and such plant use has been popular since at least the seventeenth century [50]. Thorny plants such as *C. speciosa*, *Citrus trifoliata*, *Lycium chinense*, *Eleutherococcus* sp., and *Zanthoxylum piperitum* were used in historical times [49, 50]. Along with the increased use by flower shops in eastern Japan [20], hedge use of *E. japonicus* became popular in the early twentieth century. At our study site, *D. crenata* was observed only in Oodake village, but the species was frequently used as demarcation trees in upland fields in eastern Japan [19, 20, 23]. In this study, three informants noted that those species frequently used for farmland demarcation (e.g., *C. speciosa, E. japonicus*) were also suitable as hedge plants. Moreover, many minor species used as demarcation trees along the Hijikawa River include popular gardening or hedge plants such as *H. syriacus*, *Amygdalus persica*, *O. fragrans*, *Nandina domestica*, and *Camellia japonica*. Thus, historical texts on plant use and perceptions by local people suggest that similar plants are used as demarcation trees, hedges, and gardening plants.

### Other factors influencing tree choice

Many informants (73.4%) stated that demarcation trees did not have multiple uses, but some of the trees were maintained after past commercial crop production and subsistence use were abandoned. Six informants reported that some individuals of *Morus* sp. and *Celtis sinensis* were left after their use for commercial sericulture, and *S. koriyanagi* was left after use for fiber production. Both of these industries declined in the mid-twentieth century [33, 34], suggesting that the use of some demarcation tree species has a shorter history in the region than that of *C. speciosa*. Finally, the fruits of *G. jasminoides* were traditionally used as food colorants, indicating that subsistence use is also another reason for species choice.

The natural vegetation setting is likely another factor affecting the local tree choice. *Salix pierotii* and *S. chaenomeloides* commonly grow along river banks around the study region. According to our interviews, these trees did not provide any material for tools or crops. Therefore, we presume that the local people initially started planting those *Salix* species as farmland demarcation trees because they were easy to collect from the surrounding natural habitat and easy to plant by cuttings.

### Implications for the landscape conservation

The two villages Gorou and Wakamiya along the river’s middle reach, which have been designated as an important cultural landscape by the Agency of Cultural Affairs [32], mainly included trees of Group 3 of our clustering results (Figs. 2 and 3). However, their villages’ tree composition is clearly different from those of the lower and upper reach villages. To conserve the full diversity of species used as demarcation trees in the region, villages in both the lower and upper reach areas should receive this cultural designation as well. In addition, if *C. speciosa* is indeed the prototypical demarcation tree, the lower reach areas such as Hataki and Shiba are preserving more traditional tree composition than middle reach villages. Finally, the frequent use of *Salix* species in the upper reach areas may be preserving another historical demarcation tree usage.

## Conclusions

In the Ozu region, people built stone walls and planted trees and bamboos along river banks several centuries ago as counter-measures against periodic flooding [33]. Irregular catastrophic flooding events in the region have devastated farmland on the alluvial plains, making the use of trees to demarcate farm boundaries necessary. Our research suggests that the demarcation tree composition in the region has been gradually changing over time, and species choices are driven by the ease of acquisition and transplanting, horticultural or esthetic preferences of local people, the historical trends of commercial crop production and subsistence plant use, and a possible linkage with fading folk faiths.

Even if tangible historical evidence is unavailable, information such as folk nomenclature of vegetation types, abiotic conditions of habitats, landscape units, and place names can successfully provide the ethnobiological background of various ecotopes [26]. Our study suggests that folk nomenclature of plant species used for a specific agricultural purpose along with their spatial distribution patterns and traditional knowledge can provide clues to trace chronological background of ecotopes in anthropogenic landscapes.
